# Response to Letter to the Editor: “Checkpoint Inhibitor-Associated Autoimmune Diabetes is Distinct From Type 1 Diabetes”

**DOI:** 10.1210/clinem/dgaa144

**Published:** 2020-03-19

**Authors:** Venessa H M Tsang, Rachel T McGrath, Roderick J Clifton-Bligh, Richard A Scolyer, Valerie Jakrot, Alexander D Guminski, Georgina V Long, Alexander M Menzies

**Affiliations:** 1 Department of Diabetes, Endocrinology & Metabolism, Royal North Shore Hospital, St Leonards Sydney, Australia; 2 Sydney Medical School, Northern, Faculty of Medicine and Health, The University of Sydney, Sydney, Australia; 3 Cancer Genetics Laboratory, Hormones and Cancer Group, Kolling Institute of Medical Research, Sydney, NSW, Australia; 4 Melanoma Institute Australia and The University of Sydney, Sydney, Australia; 5 Department of Tissue Pathology and Diagnostic Oncology, Royal Prince Alfred Hospital, Sydney, Australia; 6 Departments of Medical Oncology, Royal North Shore and Mater Hospitals, Sydney, Australia

## Response

We would like to thank Dr. Kotwal and Dr. Kudva for their interest in our paper and for their letter to the editor. In response to their comments, we acknowledge that the reviews published by Stamatouli et al (1) and Kotwal et al (2) both add to the literature in this area, in addition to a number of other reviews (3–5), which have all been published since our submission.

In response to the queries, all patients were followed by the oncologists for the duration of the treatment with immune checkpoint therapy, and thereafter indefinitely on a 3- to 6-month basis. We note that the authors’ review included those with worsening type 2 diabetes (T2DM), whereas we focused our cohort only on what we define as checkpoint inhibitor-associated diabetes mellitus (CIADM), where patients develop sudden new onset insulin-requiring diabetes associated with a low C-peptide. In our series, only 1 of the 10 subjects developed this after pre-existing T2DM. We did not review those who developed steroid-induced hyperglycaemia or worsening T2DM in the absence of ketosis, as these scenarios likely represent different entities, and we wished to study the purest cohort of this new CIADM entity. As this is a retrospective cohort, testing of autoantibodies is not possible on the stored serum but will be performed prospectively in future studies.

As discussed by the authors, we feel that CIADM is a novel representation of autoimmune diabetes and likely represents both a subgroup of “type 1-like” diabetes, as well as a novel entity, which is likely to have a different pathobiology yet to be determined.

## Additional Information

***Disclosure Summary:*** A.M.M. has served on advisory boards for BMS, MSD, Novartis, Roche and Pierre-Fabre. V.T. has no competing interests. G.V..L reports personal fees from Aduro, personal fees from Amgen, personal fees from Array, personal fees from BMS, personal fees from MSD, personal fees from Novartis, personal fees from Oncosec, and personal fees from Pierre Fabre outside the submitted work. R.C.B. has served on advisory boards for Eisai, Amgen, and Bayer; he has received speaking honoraria from Eisai, Amgen, Novo Nordisk, and IPSEN. A.G. has served on advisory board for BMS, Sun Pharma, Pfizer, Merck, kgA, Roche, and Eisai outside the submitted work. The corresponding author had full access to all the data in the study and had final responsibility for the decision to submit for publication.
